# A method for authenticating the fidelity of *Cryptococcus neoformans* knockout collections

**DOI:** 10.1128/spectrum.00501-25

**Published:** 2025-08-28

**Authors:** Ella Jacobs, Quigly Dragotakes, Madhura Kulkarni, Kimberly Kuhn, Ao Shen, Amanda Dziedzic, Anne Jedlicka, J. Marie Hardwick, Arturo Casadevall

**Affiliations:** 1 W. Harry Feinstone Department of Molecular Microbiology & Immunology, Johns Hopkins Bloomberg School of Public Health 25802 , Baltimore, Maryland, USA; Virginia-Maryland College of Veterinary Medicine, Blacksburg, Virginia, USA

**Keywords:** genetics, fungi, *Cryptococcus neoformans*, deletion collection, resource

## Abstract

Gene knockout (KO) strain collections are important tools for discovery in microbiology. *Cryptococcus neoformans*, a human fungal pathogen, has an available genome-wide gene deletion collection that is widely used by the research community. We uncovered mix-ups in the assembly of the commercially available *C. neoformans* deletion collection of ~4,700 unique strains acquired by our laboratory. Evidence supporting a mix-up includes RNAseq analysis that identified transcripts for the gene listed as the KO. The mystery was soon solved as this same KO strain lacked RNA transcripts for a different KO strain gene found in the same plate position in an earlier partial KO collection, suggesting a plate swap between two KO collections. Therefore, we developed a quick PCR assay to distinguish the two KO collections based on the size differences between their nourseothricin (NAT)-resistance cassettes, confirmed by genome sequencing. Here, we report that nine of the first 15 plates of the 42-plate our KN99ɑ KO collection had been replaced with the corresponding plates from an earlier partial KO collection. We provide additional evidence that the remaining plates are correct, and the simple authentication method presented here serves as a quick check to identify similar mix-ups in the KO collections.

**IMPORTANCE:**

Gene KO strain collections are important tools for discovery in microbiology. The human fungal pathogen *Cryptococcus neoformans* has an available genome-wide deletion collection that is widely used by the research community. Here, we report that our KN99ɑ collection is comprised of mixed plates from two independent KO libraries and present a simple authentication method that other investigators can use to distinguish the identities of these KO collections. Above all, this article serves as a reminder to users of the 2015 KO library collection to screen the plates before undertaking large phenotyping experiments.

## INTRODUCTION

In the field of microbiology, genome-wide gene knockout (KO) collections are important investigative tools to rapidly screen for genes associated with specific phenotypes. For studies of *Cryptococcus neoformans*, several KO collections are available (1, 2), including the genome-wide knockout (KO) collection containing ~4,700 strains generated in the Madhani laboratory, an arduous feat compared to the more tractable model yeast *Saccharomyces cerevisiae* (3–5).

The first Madhani *C. neoformans* gene KO collection was generated in the H99W background strain (2008 collection of ~1,300 KO strains) (6). H99W, also known as “Wimp,” is a laboratory-adapted strain with attenuated virulence and melanization defects attributed to a 7 bp insertion in the *LMP1* gene (7, 8). Given the severe virulence attenuation of the H99W strain, a subsequent genome-wide KO collection was generated in the virulent KN99α background strain, arrayed in 96-well plates and distributed in three phases, designated as 2015 (22 plates), 2016 (20 plates), and 2020 (7 plates) collections containing one KO strain per well (9). KO strains were generated by gene replacement/disruption via homology-directed repair using gene-specific ~1 kb homology arms flanking a nourseothricin acetyl transferase (NAT) cassette (9).

Our 2015 and 2016 KO collections were purchased from the Fungal Genetics Stock Center (FGSC) in March 2018 and the 2020 KO collection in March 2021. These purchased collections have proved useful for screening and identifying genes associated with non-lytic exocytosis (10), protein secretion (11), and cell death (12). We utilized this KO library to phenotypically screen selected KO strains to follow up on proteomics studies. During routine PCR confirmation across the locus where gene replacement was engineered, we realized that many strains from our 2015 KO collection did not contain the expected gene disruption, despite being NAT resistant.

We tested plates of our 2015 KO collection and found that rather than individual well mix-ups or well-well contamination, whole plates from the KN99 KO appear to have been swapped with 2008 KO collection plates. The 2008 KO collection contains strains that are arrayed in 14 96-well plates. Based on the strains we tested, only plates within our first 14 plates of the 2015 KO collection were impacted. Overall, a total of nine non-consecutive plates from the 2008 KO collection appeared to be directly swapped into the 2015 KO collection, while no mix-ups were detected for the strains tested from the 2016 and 2020 KO collections. We developed a simple, cost-effective method of testing KO strains purchased from the Fungal Genetics Stock Center (FGSC) by comparing NAT cassette sizes. However, locus-specific validation is required to fully confirm plate integrity. This protocol will allow screening to quickly assess potential fidelity issues for follow-up.

## RESULTS

### Generation of primers to reveal the location and presence of NAT cassettes within our 2015 KO collection strains

**Fig 1:**
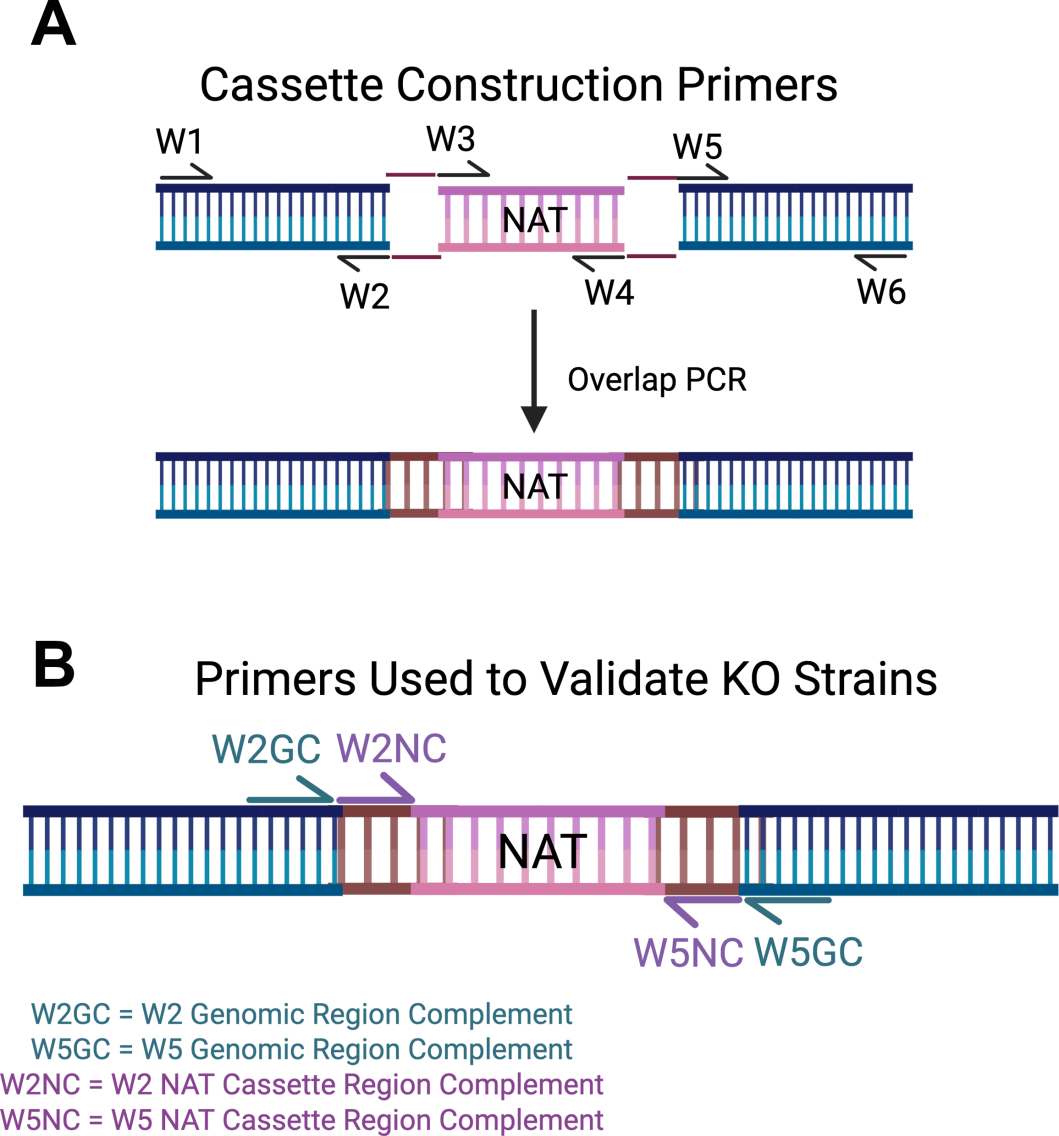
Primer design schematics for KO construction and validation**.** (**A**) Deletion cassette construction was created based on the information in reference 9: W1, W2, W5, and W6 primers are publicly available for each strain on the FGSC website. W3 and W4 primers are not publicly available. Created with BioRender. (**B**) Strain verification schematic. The reverse complement of the genomic region of W2 (W2GC) and W5 (W5GC) was used to confirm deletion fidelity at each locus.

Using overlap PCR, the Madhani lab constructed KO cassettes containing the NAT resistance gene plus 1 kb flanking homology arms. These cassettes were subsequently transformed and inserted into the *C. neoformans* gene of interest by homology-directed repair (HDR) (9). The primers used by the Madhani lab to generate the 2015 KO collection are available on the FGSC website (Fig. 1A). Primer pairs W1 + W2 and W5 + W6 amplify the flanking 5’ and 3’ recombination arms, respectively. Primer pairs W3 + W4 (not included in the available primer data set) were used to amplify the NAT resistance cassette, which was fused to the genomic recombination arms (Fig. 1A).

We generated two types of primers to verify either the location or presence of the NAT drug selection marker in our copy of the KO collections (Fig. 1B; Table 1 and 2). To amplify the NAT cassette in any KO strain, regardless of location, we generated primers complementary to the 5′ of W2 +W5 NAT cassette region, designated as W2 NAT Complement (W2NC) and W5 NAT Complement (W5NC) (Fig. 1B, Table 1). According to the FGSC spreadsheet, the 5′ end of the W2 + W5 primer pairs is the same across all 2015 KO constructs. Therefore, this primer pair could be used on any KO strain to amplify the NAT cassette as it contains no locus-specific base pairs.

**TABLE 1:** NAT cassette PCR primers

Region	NAT cassette primer (5′→3′)
W2 NAT region RC (W2NC)	gttggatccgctgctaggcgcgccgtg
W5 NAT region RC (W5NC)	gttggatgcagggatgcggccgctgac

**TABLE 2:** W2GC and W5GC locus-specific primers

CNAG	2015 plate	2015 well	Locus-specific F primer (W2GC)	Locus-specific R primer (W5GC)
CNAG_06104∆	0	B8	CTGCATCCCCATCCATCACATC	GGTCAGTGACTTTCGACATCTC
CNAG_02179∆	2	H12	AACATCGTATAGTACAGCAAAC	AAAATTCCGTGGGAATATGTAA
CNAG_02801∆	2	B7	CCACATCACACAAGACATCAAA	CATGCACGATCAATCTATGAAA
CNAG_04981∆	3	D4	CTGCGATTCTAAGCCGGTCAAT	AACATTCCTTGAAATCAACCGC
CNAG_01019∆	4	A1	TCAGGAACTCTATCTAATCGAA	CGAATATGTAGTAACGGTGCGC
CNAG_04891∆	5	A10	GAATCAAATAATAGACCATACACTTACAGTCATA	CACACTCTCCTCCGAGCATCAT
CNAG_01047∆	8	H4	ACGAGCTAGAATCATATAAGTTCAGAAAC	ACAATCCACTAACCGATCAAGG
CNAG_03019∆	8	H6	CTGTATTCTGCCCTAACCCG	CAACAATTTCCTAACGGTGACC
CNAG_04388∆	10	A4	TTACACAGATCTCACTTCCAAA	GTTTACACATTGTTCATAAAGC
CNAG_04321∆	11	D9	ATTACTATCCATTCATCCTACA	TTCCCAAAGCAACAAGACTAGC
CNAG_07638∆	12	D1	ATCCCATACGCCCCAGCAAGAA	TACACATTGCAAGAAGACAGAAAAAAAAAGTGACT
CNAG_02503∆	15	G7	TTACATTCCACACTAATCCAAC	CTCCATATTACAGCTAATGCCT
CNAG_05893∆	17	A10	CATTACACTCGCACTCGCCATA	ACGGAATTATAATCTCTTCTAGAGAGAC

To verify the location of the NAT cassette in the correct KO gene locus, we generated primers that bind the genome immediately flanking the NAT cassette. These primers were adapted from W2 and W5 sequences, complementary to the 3′ 27 nucleotides of primers W2 and W5, indicated as W2 Genomic Complement (W2GC) and W5 Genomic Complement (W5GC) (Fig. 1B; Table 2). The sequence of W2GC and W5GC differs for each gene.

### Targeting genomic loci of our 2015 KO collection library strains via PCR

**Fig 2:**
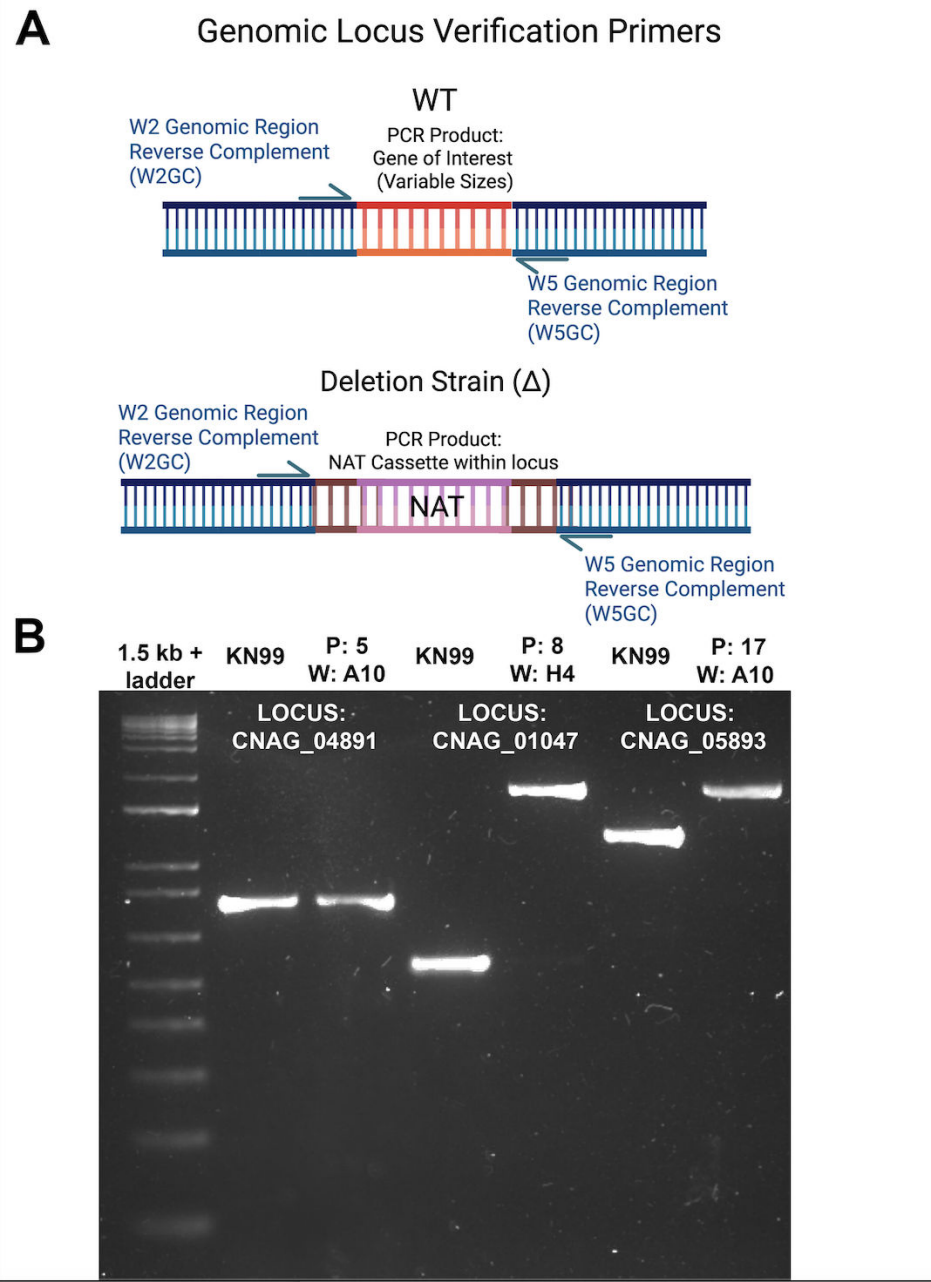
PCR validation of KO collection strains targeting the genomic locus. (**A**) In WT KN99, these primer pairs amplify the gene of interest. In KO strains, these primer pairs will only amplify the NAT cassette. (**B**) Example genomic locus strain verification 1.5% agarose gel using the methodology in panel B. Primers targeted the reverse complement of the genomic locus-specific sequence in the W2 (W2GC) and W5 (W5GC). K-denoted lanes contain WT (KN99) PCR products, and ∆-denoted lanes contain putative deletion strain PCR products. Numbers refer to the CNAG locus corresponding to the W2 W5 sequence location. CNAG_04891*∆* did not contain a NAT cassette within CNAG_04891, as the WT and deletion strain PCR products are the same size. Both CNAG_01047*∆* and CNAG_05893*∆* contain NAT cassettes in the expected locus.

W3GC and W4GC primer sets for 13 of genes were used to amplify genomic DNA isolated from both WT and KO strains in the 2015 KO collection (Fig. 2A; Table 3), and the sizes of amplified products were compared on agarose gels (Fig. 2B). We expected the genomic PCR products amplified from the KO strains to differ in size from the WT genomic PCR products. However, the genomic PCR products from 5 out of the 12 putative KO strains were the same size as the WT gene products, such as CNAG_04891 (Fig. 2B). This suggested that despite NAT resistance, the expected gene was not replaced. By contrast, other KO strains from the 2015 KO collection appeared to contain the same size NAT cassette (1,743 bp), which differed from the expected sizes of their respective WT gene, for example, CNAG_01047 and CNAG_05893 (Fig. 2B). It is worth noting that this methodology only works if the gene of interest is different in size from the NAT cassette. If not, further validation with a diagnostic restriction enzyme digestion, alternative primer design, or sequencing of the amplified products is necessary.

**TABLE 3:** 2015 KO collection strains tested using locus-specific primers^*a*^

CNAG	Plate, well	WT product size	Experimental product size	Conclusion	Plate status
CNAG_06104	0, B8	2,258 bp	1,743 bp	KO present	Expected plate
CNAG_02179	2, H12	3,088 bp	3,088 bp	KO absent	Plate mix-up
CNAG_02801	2, B7	524 bp	524 bp	KO absent	Plate mix-up
CNAG_04981	3, D4	2,618 bp	2,618 bp	KO absent	Plate mix-up
CNAG_01019	4, A1	980 bp	980 bp	KO absent	Plate mix-up
CNAG_04891	5, A10	781 bp	781 bp	KO absent	Plate mix-up
CNAG_01047	8, H4	547 bp	1,743 bp	KO present	Expected plate
CNAG_03019	8, H6	3,338 bp	1,743 bp	KO present	Expected plate
CNAG_04388	10, A4	1,002 bp	1,743 bp	KO present	Expected plate
CNAG_04321	11, D9	2,597 bp	1,743 bp	KO present	Expected plate
CNAG_07638	12, D1	1,451 bp	1,743 bp	KO present	Expected plate
CNAG_02503	15, G7	882 bp	1,743 bp	KO present	Expected plate
CNAG_05893	17, A10	1,206 bp	1,743 bp	KO present	Expected plate

^*a*^ NT indicates not tested. KO present indicates the strain contained the expected gene replacement. KO absent indicates the strain did not contain the expected replacement.

### Larger NAT cassette size correlated with strains lacking the expected KO

**Fig 3:**
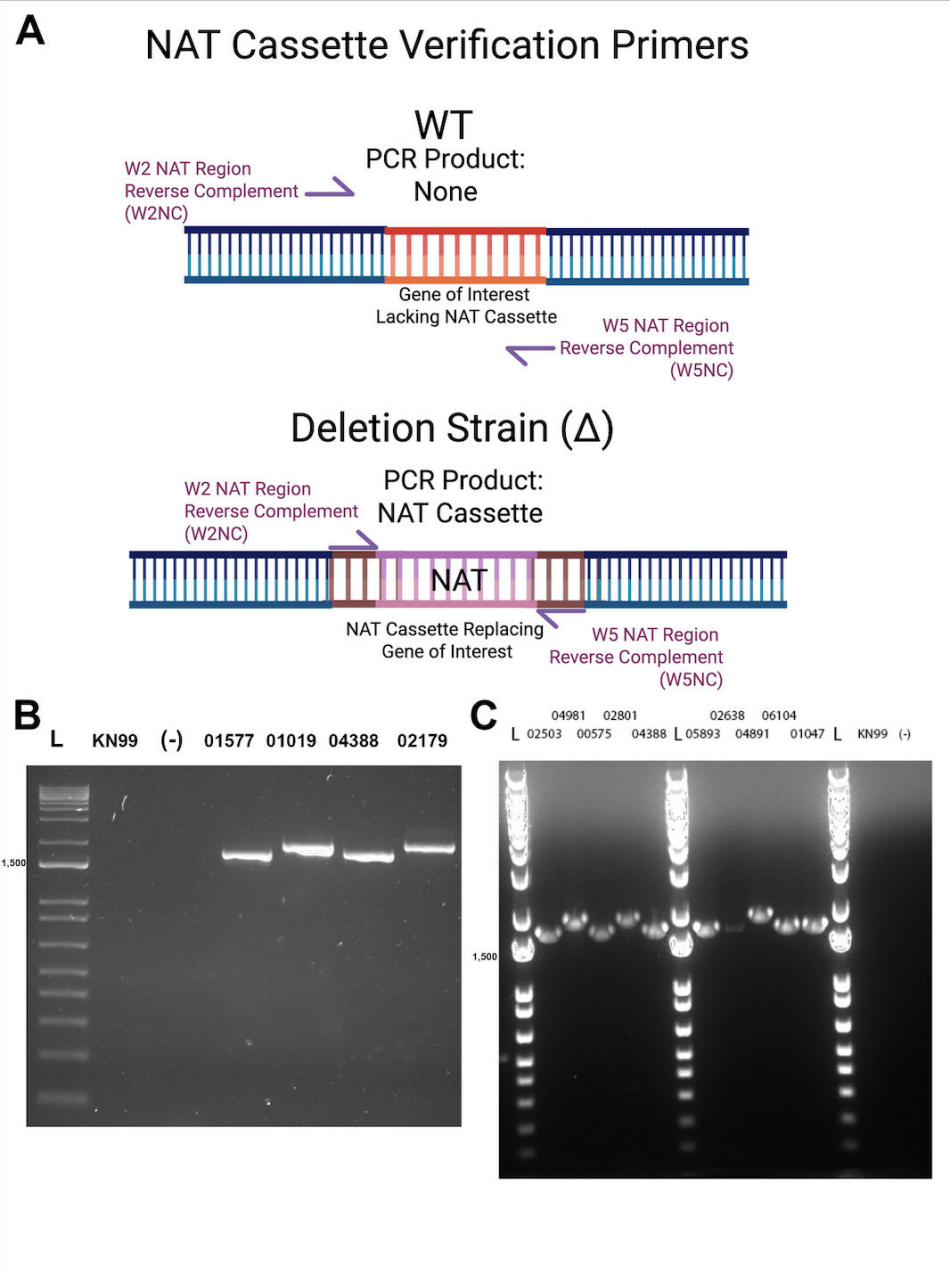
Comparing NAT cassette sizes between KO strains reveals size differences that correlate with locus-specific replacement failures. (**A**) NAT cassette verification methodology. The reverse complement of the NAT cassette-specific region of W2 (W2NC) and W5 (W5NC) was used to confirm the presence of the NAT cassette in putative KO strains. In WT KN99ɑ, these primer pairs will not amplify a product due to a lack of a replacement cassette. In KO strains, these primer pairs will only amplify the NAT cassette in any KO strain, as there are no genomic-locus-specific base pairs. (**B**) NAT cassette size comparisons. KN99ɑ and a no-template control show a lack of amplification. NAT cassettes PCR products from CNAG_02179*∆* and CNAG_01019*∆,* two strains that failed to have expected locus-specific disruptions, were larger in size than the KO strains with correct KOs. (**C**) Amplification of NAT cassette sequences by PCR across KO strains. This further confirmed that the larger NAT cassette size correlated with locus-specific PCR failures. Note that the larger NAT cassette in CNAG_04981*∆* corresponds to the locus-specific PCR failure in Fig. 2B. Both CNAG_01047*∆* and CNAG_05893*∆* contain smaller NAT cassettes and passed the PCR test from Fig. 2B. We found the differences in NAT cassette size to be predictive of a KO strain’s locus-specific failure.

To verify the presence of a NAT cassette in strains lacking a disruption at the expected locus, such as CNAG_04891∆*,* we used the W2NC + W5 NC primer pairs discussed in Fig. 1B and Table 1. This primer pair is capable of hybridizing to the 5′ and 3′ ends of the NAT cassette (complementary to the 5′ 22-nucleotides of primers W2 and W5), which are common to all the KO strains (Fig. 3A). As expected, no NAT cassette was amplified from WT KN99ɑ, while all KO strains yielded a PCR product, including strains that did not contain the expected KO (Fig. 3B and C).

There were two potential explanations for our finding that all strains lacking expected gene KOs still possessed NAT cassettes within the genome. The first being that the cassettes were replacing other regions of the genome, and the second being that KO strains were mixed up. An important clue favoring a KO strain mix-up emerged by comparing NAT cassette sizes between strains using primers W2NC + W5 NC (Fig. 3B and C; Table 4). We found that strains lacking the expected gene KO (CNAG_01019∆, CNAG_02179∆, CNAG_04981∆, CNAG_02801∆, and CNAG_04891∆) had ~2 kb NAT cassettes. By contrast, strains with the expected gene KO had 1,743 bp-sized NAT cassettes. We wondered if the differences in NAT cassette size could be due to barcoding or other potential changes that would differ between KO collections. Importantly, the 2008 KO collection cassettes are barcoded (6).

**TABLE 4:** 2015 KO collection strains tested for NAT cassette size differences

CNAG	Plate, well	NAT cassette size	Plate status
CNAG_06104	0, B8	1,743 bp	Expected plate
CNAG_02435	1, E7	2 kb	Plate mix-up
CNAG_02179	2, H12	2 kb	Plate mix-up
CNAG_02801	2, B7	2 kb	Plate mix-up
CNAG_04981	3, D4	2 kb	Plate mix-up
CNAG_01019	4, A1	2 kb	Plate mix-up
CNAG_04891	5, A10	2 kb	Plate mix-up
CNAG_02685	6, D9	2 kb	Plate mix-up
CNAG_04122	7, C6	2 kb	Plate mix-up
CNAG_01047	8, H4	1,743 bp	Expected plate
CNAG_03019	8, H6	1,743 bp	Expected plate
CNAG_00306	9, F10	2 kb	Plate mix-up
CNAG_04388	10, A4	1,743 bp	Expected plate
CNAG_04321	11, D9	1,743 bp	Expected plate
CNAG_07638	12, D1	1,743 bp	Expected plate
CNAG_02489	13, F7	1,743 bp	Expected plate
CNAG_05592	14, A10	2 kb	Plate mix-up
CNAG_02503	15, G7	1,743 bp	Expected plate
CNAG_03729	16, F1	1,743 bp	Expected plate
CNAG_00064	18, G7	1,743 bp	Expected plate
CNAG_06384	19, F6	1,743 bp	Expected plate
CNAG_00006	20, C4	1,743 bp	Expected plate
CNAG_00308	21, A8	1,743 bp	Expected plate

### mRNA-Seq and DNA-Seq evidence to support a direct plate swap between the 2015 and 2008 KO collections

**Fig 4:**
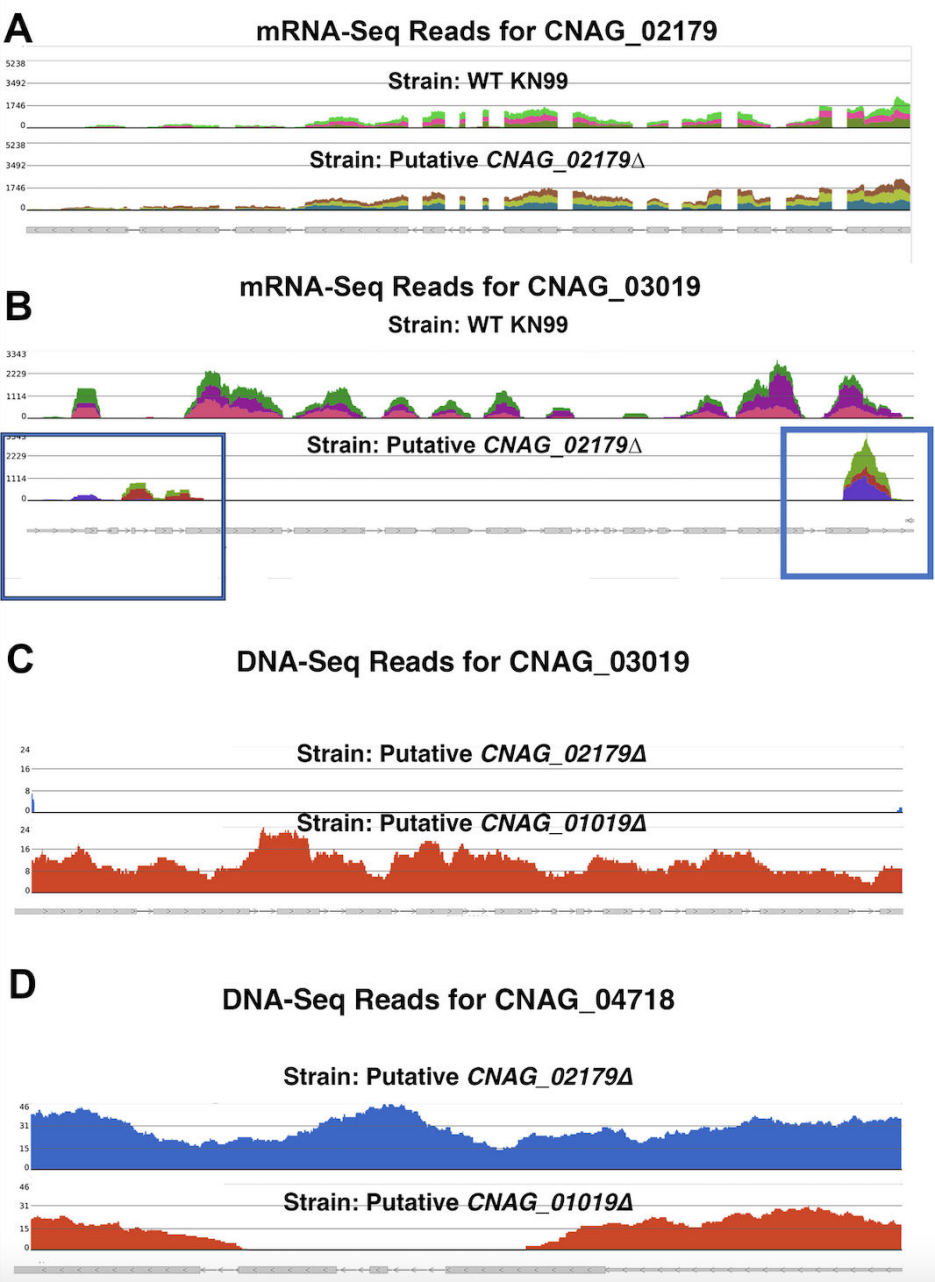
mRNA-Seq and DNA-Seq reads in the putative *CNAG_02179∆* and *CNAG_01019∆* strains show that both strains have disruptions in the 2008 plate expected KO instead of the 2015 expected KO. (**A**) mRNA-Seq reads comparing the coverage of CNAG_02179 in the WT KN99 strain (top) to the strain in our 2015 library plate 4 well H12 strain (putative CNAG_02179*∆*) (bottom). Expression levels were identical in the WT and KO strain, confirming no disruption in the expected locus. (**B**) CNAG_03019 is the actual KO gene in putative *CNAG_02179∆*. The mRNA-Seq reads comparing the coverage of CNAG_03019, the gene that would be KO if the 2015 collection plate was mixed up with a 2008 collection plate 4, well H12 strain. The top reads are from WT, and the bottom reads are from plate 4, well H12 strain (putative *CNAG_02179∆*). Boxes represent the gene replacement location specific to the 2008 version of the CNAG_03019 KO. In the 2015 version of the KO, the entire ORF is replaced. (**C**) DNA-Seq reads confirm the disruption of CNAG_03019 in the putative CNAG_02179*∆* strain. (**D**) DNA-Seq reads show the disruption of CNAG_04718 in the putative *CNAG_01019∆* strain, the gene that would be disrupted if the 2015 collection plate was mixed up with a 2008 collection plate.

We wanted to test whether the 2015 KO library plates were mixed up with 2008 KO library plates. To do this, we looked at the 2008 library plate map, available in the supplemental information of the accompanying paper (6). We used data from a previous RNAseq experiment conducted on putative CNAG_02179∆ to confirm the plate swap (Fig. 4A and B). CNAG_02179∆ was pulled from plate 2, well H12 from our 2015 labeled library plates. CNAG_03019∆ is the strain in plate 2, well H12 in the 2008 KO collection. Importantly, compared to the 2015 KO collection, the 2008 KO collection often used different homology arm constructs. Therefore, comparing the KO location would add a layer of evidence that these strains came from a 2008 KO collection. As such, the 2008 version of the CNAG_03019 KO has two regions within the ORF which are not replaced: an additional ~500 bp on the 5′ end and ~100 bp on the 3′ end. By contrast, the 2015 version of the CNAG_03019 KO replaces the entire ORF. In agreement with a library mix-up, the location of mRNA-Seq reads within the CNAG_03019 KO locus of putative *CNAG_02179∆* mirrors the expected replacement location of the 2008 CNAG_03019 KO strain. RNAseq analysis provided definitive evidence demonstrating the presence of transcripts from the putative KO locus of CNAG_02179 and the absence of transcripts from the gene CNAG_03019, found in the analogous plate position in the 2008 KO collection. We confirmed these results with DNA-Seq performed on the putative *CNAG_02179∆* (Fig. 4C). Furthermore, we performed DNA-Seq on the putative *CNAG_01019∆* strain from plate 4 well A1, which also contained a ~2 kb NAT cassette. If plate 4 of the 2015 collection was mixed up with plate 4 of the 2008 library collection, then well A1 would contain a strain with a disruption in CNAG_04718. In agreement with a plate mix-up occurring, we found that the putative *CNAG_01019∆* strain possesses a disruption in CNAG_04718 instead (Fig. 4D).

### PCR-based evidence for a direct plate swap between the 2015 and 2008 KO collections

**Fig 5:**
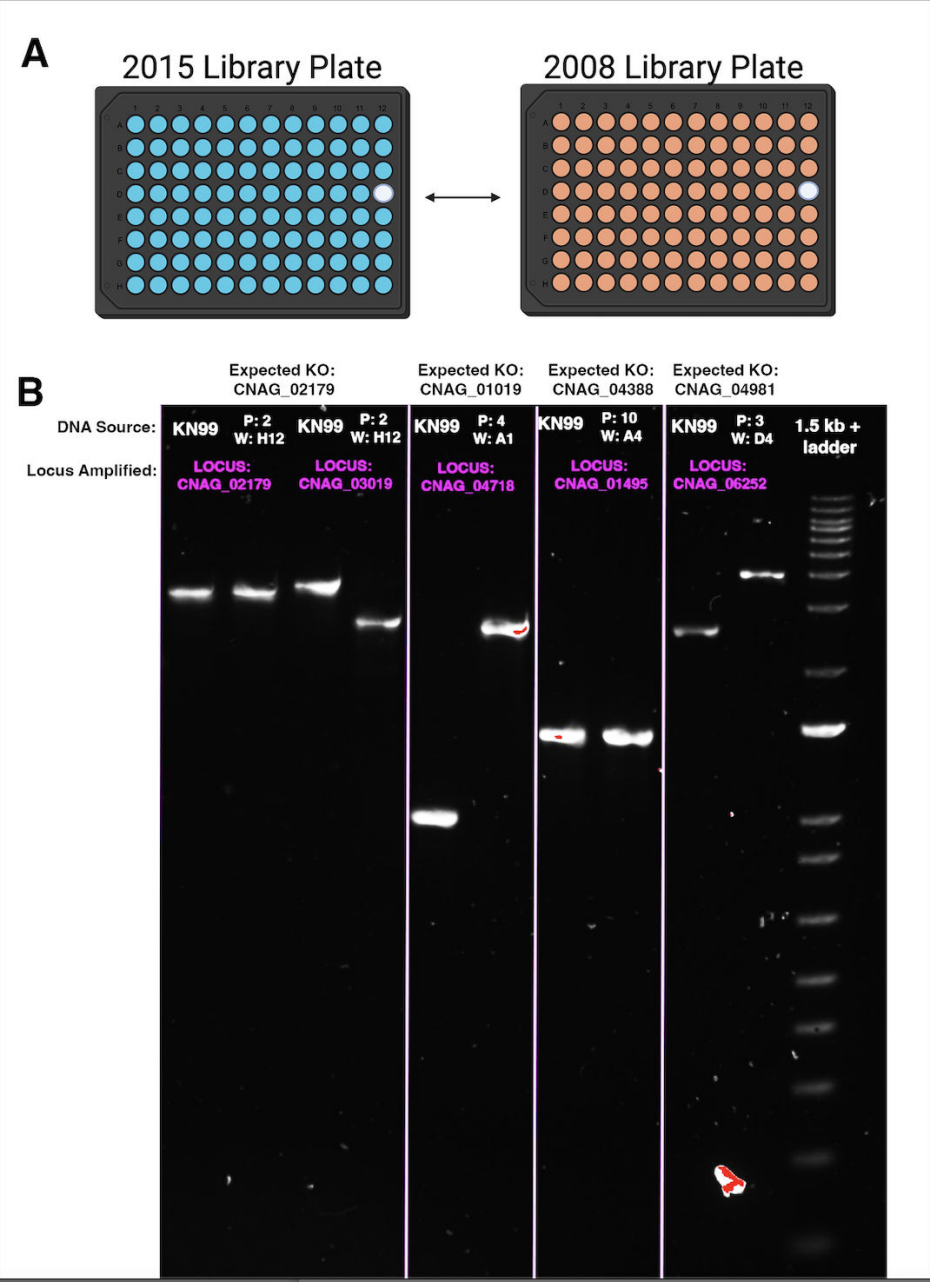
Summary PCR and confirmation of 2015 and 2008 direct plate swaps. (**A**) Based on our findings, the whole 2015 plates were directly swapped with the 2008 plates, not individual wells; (**B**) 1.5% agarose gel comparing PCR product sizes (1 kb + ladder). All lanes contain PCR products amplified using the locus-specific strategy outlined in Fig. 1 and 2. Expected KO indicates the CNAG that should be disrupted in the 2015 strain. Individual lane labels (white text) indicate genomic DNA source (KN99ɑ WT or 2015 plate and well position of the strain). Locus labels (magenta text) indicate the gene amplified by the W2GC + W5 GC primer pairs. Of these strains, plate 2, plate 4, and plate 3 contained disruptions in the 2008 CNAG loci instead of the 2015 loci. The strain from plate 10 did not contain a disruption in the 2008 locus.

The evidence thus far suggested that some plates assembled into our copy of the 2015 KO collection were instead derived from another KO collection, most likely the earlier 14-plate KO collection on the avirulent H99W background from 2008 (Fig. 5A). We followed up the RNAseq findings in Fig. 4 with PCR-based experiments using the W2GC + W5 GC primer pairs for the expected genes if the 2015 plates were swapped with 2008 plates (Fig. 5B). It appeared that the same universal primer sequences were apparently used for both 2008 and 2015 KO collections. In agreement with the mRNA-Seq results, the putative CNAG_02179∆ strain found in plate 2, well H12 does not contain a KO at CNAG_02179. Instead, the strain contains a KO in CNAG_03019, the KO found in the analogous plate position in the 2008 KO collection. In addition, as described above, the W2GC and W5GC primers from the 2015 KO collection sequences were used to amplify the suspected 2008 swapped KO strains (Fig. 5B). The PCR product amplified targeting the CNAG_03019 KO locus within the putative CNAG_02179∆ strain is larger than the NAT cassette alone, in agreement with the RNAseq results and supporting the hypothesis that the strain is of 2008 KO collection origin.

To confirm that the mix-up occurred in other plates, we tested two other strains that we found lacked the expected KO and possessed a ~2 kb NAT cassette (plate 4 well A1 and plate 3 well D4). These two strains also contained KOs in the 2008 CNAG loci instead of the 2015 loci. By contrast, we tested the strain in plate 10, well A4, which we previously found contained the expected KO at CNAG_04388 and a 1,743 bp NAT cassette. The strain from plate 10 did not contain a disruption in the 2008 KO collection locus. For locus-specific confirmation, 2008 KO collection-specific primers could be used, but we feel the size differences add further evidence to confirm the KO is in fact of 2008 origin. Ultimately, we discovered that the larger NAT cassette size may be the result of a direct plate swap between the 2015 and 2008 KO collections.

### Our 2015 KO collection plates 1–7, 9, and 14 are 2008 KO collection plates

**Fig 6:**
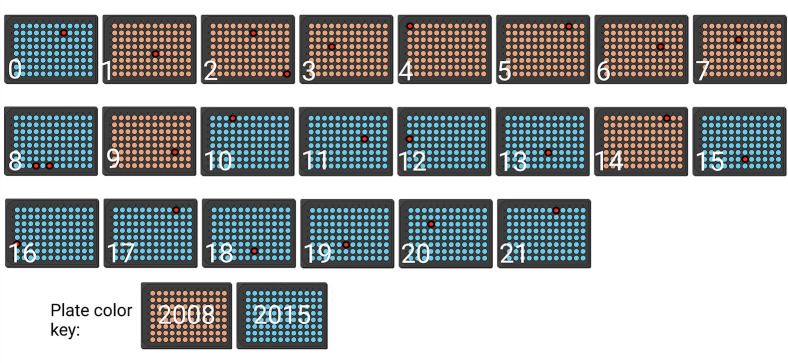
Plates 1–7, 9, and 14 are directly mixed up with 2008 KO collection plates. Of the 22 plates in our 2015 KO collection, only the first 14 plates were impacted. Specifically, plates 1, 2, 3, 4, 5, 6, 7, 9, and 14 were directly swapped. Orange plates indicate 2008 swapped plates. Blue plates indicate 2015 library plates. Red wells indicate tested wells (see Tables 3 and 4).

Given the fact that some strains possess the anticipated 2015 gene KO while others contain a 2008 KO, the mixed-up KO strains are restricted to a subset of 96-well plates in the 2015 KO collection. We systematically PCR tested 24 strains across all 22 plates in our 2015 library using a mix of the genomic locus and NAT cassette size strategy (Fig. 6; Tables 3 and 4). Initially, strains were selected based on our interest in gene candidates identified from proteomics experiments. Strains in all untested plates were randomly selected. The most parsimonious interpretation of our results is that whole plates were directly swapped, not individual wells. The 2008 KO collection possesses 14 plates, and only plates within our first 14 plates of the 2015 KO collection were impacted. Plates #1–7, 9, and 14 of our 2015 KO collection are instead KO strains from the 2008 KO collection, while plates #0, 8, 10–13, and 15–21 are the expected 2015 KO collection plates. The PCR methodology outlined in this work offers a quick, cost-effective methodology for testing the presence of a plate swap within a 2015 KO collection.

## DISCUSSION

Here, we describe an unfortunate plate mix-up involving cryptococcal gene KO libraries and demonstrate a cost-effective, time-saving method for authenticating the fidelity of (2) KO collections. This will allow other labs to rapidly determine the status of their 2015 libraries. Using NAT cassette-specific primers, not locus-specific primers, for size comparison screening of the library proved a useful tool for initial screening. However, we did not test every strain, and other sized NAT cassettes may exist within the library. Follow-up with locus-specific testing is essential to confirm that plates were swapped.

There are several limitations to the methods presented here. First, we have found that locus-specific primers were not specific enough to compare replacement cassette sizes between 2008 and 2015 KO strains. The same replacement sites were not used between the 2008 and 2015 KO libraries to generate the same KO strains. Hence, care must be used when applying locus-specific primers to suspected 2008 swapped strains, as PCR product sizes will differ. An example of this can be seen when CNAG_03019∆ primers from the 2015 KO library are applied to the CNAG_03019∆ from the 2008 swapped KO library plate. To confirm a 2008 KO library swapped plate in a locus-specific manner, we recommend using 2008 KO library locus-specific primers. Alternatively, be aware of the expected size differences when performing the experiment. We found that a 1.5% agarose gel was most appropriate to resolve the size differences between NAT cassettes. Finally, we have no information on where, when, or how the plate swap occurred, but we are certain that it occurred prior to the arrival of the library in the Casadevall laboratory since we have original plates that served as the point of origin of the mutated strains used here. We reached out to the FGSC and ascertained that our plates were sent by a previous employee who is no longer in contact with the stock center. Preventive actions have been taken to physically separate the libraries into different freezers to ensure future mix-ups will be impossible. Furthermore, plates are being individually tested with PCR to ensure plates are correctly labeled. Our advice to the community is to use the methods described here to check their libraries before using the mutants for large screening experiments.

In summary, our experience is a cautionary tale of the foibles that can accompany the use of community resources like KO libraries. Although these resources are powerful tools for discovery, one must always maintain a certain skepticism when using them and validate any important finding by independent methods. Currently, these KO libraries are in many laboratories studying *C. neoformans* and continue to be useful tools for research. We are hopeful that our experiences and the validation roadmap described here will allow other laboratories to maximize their potential while avoiding false leads.

## MATERIALS AND METHODS

### Strains

Our lab purchased the 2015 and 2016 Madhani KO collections in March 2018 and the 2020 plate KO collections in March 2021.

### Genomic DNA isolation and colony PCR

Genomic DNA isolation was performed using the CTAB isolation method (13, 14). Strains were cultured in 50 mL YPD, centrifuged, and frozen overnight at 80°C. The frozen cell pellet was powdered using 0.5 mm disruption glass beads (Biospec) and an MP FastPrep-24 bead beater at a speed of 6.5 m/s in 30 s intervals until the pellet was fully pulverized. 10 mL CTAB extraction buffer (100 mM Tris, pH 7.5, 0.7 M NaCl, 10 mM EDTA, 1% CTAB, 1% beta-mercaptoethanol) was added to the culture pellet and vortexed. After 30 min of incubation at 65°C, tubes were cooled in ice water, and an equal volume of chloroform was added. The solution was mixed by inversion for 1 min and centrifuged for 10 min at max speed (RT). The top layer was transferred to a clean tube, and an equal volume of isopropanol was added. The solution was mixed by inversion for 1 min and centrifuged for 5 min at max speed (RT). The supernatant was decanted off, and 1 mL of fresh 70% EtOH was added to the sample. After 5 min of centrifugation, the supernatant was decanted off, and the pellet was resuspended in 100 µL sterile molecular biology water. For further testing of the entire library, we utilized colony PCR methodology (15). In this method, an overnight agar culture was prepared for each strain, and a sterile 10 µL pipette tip was used to streak fungal cells directly into a PCR tube. Tubes were microwaved for 2 min to release DNA. PCR mastermix and primers were added directly to the tube containing microwaved fungal cells. Phusion PCR mastermix was used to amplify genomic loci and NAT cassettes using the primer pair in Table 1. Due to the high annealing temperature of the NAT cassettes, we utilized the two-step PCR protocol, where the annealing and extension times are combined, as outlined in the Phusion manual. All reactions were run on a 1.5% agarose gel.

### mRNA-Seq

Putative CNAG_02179∆ was cultured in 1 mL YPD overnight, washed twice with PBS, and brought to a starting cell density of 1 × 10^6^ cells/mL in 10 mL minimal media. 1 mL of cells was collected at 48 h per 3 biological replicates. Yeasts were frozen in 250 µL Trizol at −80°C for future analysis. The yeasts were brought up to 1 mL of Trizol and homogenized in the FastPrep 24 (MP Bio) with Lysing Matrix C Fast Prep tubes at speed 6 for 30 s, 4 times. Homogenates were held on ice between each cycle. After homogenization, RNA extraction was performed using the PureLink RNA Mini kit with on-column DNase treatment (ThermoFisher). Quantitation of total RNA was performed with the Qubit RNA High Sensitivity Assay Kit and Qubit Flex Fluorometer (ThermoFisher), and quality assessment was performed by High-Sensitivity RNA ScreenTape analysis on an Agilent TapeStation 4200. mRNA-Seq Libraries were prepared using the Universal Plus mRNA-Seq Library prep kit (Tecan Genomics), incorporating unique dual indexes. Libraries were assessed for quality by High-Sensitivity D5000 ScreenTape on the 4200 TapeStation (Agilent Technologies). Quantification was performed with NuQuant reagent and by Qubit dsDNA High-Sensitivity Assay Kit, on Qubit 4 and Qubit Flex Fluorometers (Tecan Genomics/ThermoFisher). Libraries were diluted and an equimolar pool was prepared, according to the manufacturer’s protocol for appropriate sequencer. An Illumina iSeq Sequencer with iSeq100 i1 reagent V2 300 cycle kit was used for the final quality assessment of the library pool. For deep mRNA sequencing, a 200-cycle (2 × 100 bp) Illumina NovaSeq 6000 S1 run was performed at Johns Hopkins Genomics, Genetic Resources Core Facility, RRID:SCR_018669. mRNA-seq data were analyzed with Partek Flow NGS Software as follows: pre-alignment QA/QC; alignment to *C. neoformans* Reference Index using STAR 2.7.8a; post-alignment QA/QC; quantification of gene counts to annotation model (Partek E/M); filter and normalization of gene counts; identification and comparison of differentially expressed genes with GSA (gene-specific analysis). mRNA-seq data were aligned to *C. neoformans* using Bowtie 2, and chromosome view was used to visualize aligned reads to the putative CNAG_02179∆ strain vs WT KN99 strain (Partek Flow). The CNA3 genome assembly was used as the reference genome (NCBI RefSeq assembly GCF_000149245.1_CNA3).

## Data Availability

All sequence files and sample information have been deposited at the NCBI Sequence Read Archive, NCBI BioProject: PRJNA1194623.
